# Degradation and energy performance evaluation of mono-crystalline photovoltaic modules in Egypt

**DOI:** 10.1038/s41598-023-40168-8

**Published:** 2023-08-11

**Authors:** Doaa M. Atia, Amal A. Hassan, Hanaa T. El-Madany, Aref Y. Eliwa, Mohamed B. Zahran

**Affiliations:** Electronics Research Institute, Cairo, Egypt

**Keywords:** Engineering, Electrical and electronic engineering, Photovoltaics

## Abstract

Degradation reduces the capability of solar photovoltaic (PV) production over time. Studies on PV module degradation are typically based on time-consuming and labor-intensive accelerated or field experiments. Understanding the modes and methodologies of degradation is critical to certifying PV module lifetimes of 25 years. Both technological and environmental conditions affect the PV module degradation rate. This paper investigates the degradation of 24 mono-crystalline silicon PV modules mounted on the rooftop of Egypt's electronics research institute (ERI) after 25 years of outdoor operation. Degradation rates were determined using the module's performance ratio, temperature losses, and energy yield. Visual inspection, I–V characteristic measurement, and degradation rate have all been calculated as part of the PV evaluation process. The results demonstrate that the modules' maximum power (${P}_{max}$) has decreased in an average manner by 23.3% over time. The degradation rates of short-circuit current (${I}_{sc}$) and maximum current (${I}_{m}$) are 12.16% and 7.2%, respectively. The open-circuit voltage (${V}_{oc}$), maximum voltage (${V}_{m}$), and fill factor ($FF$) degradation rates are 2.28%, 12.16%, and 15.3%, respectively. The overall performance ratio obtained for the PV system is 85.9%. After a long time of operation in outdoor conditions, the single diode model's five parameters are used for parameter identification of each module to study the effect of aging on PV module performance.

## Introduction

The energy issue continues to be crucial for society's social and economic advancement^1^. Environmental issues arise from using nonrenewable fuel sources, especially with the rising cost of oil and the negative effects of burning fossil fuels on the environment. Different renewable energy sources today provide enough flexibility and dependability across a wide range of technologies to minimize the energy shortage as a result of rising demand^1,2^. Today, solar energy inhabits a significant position in the market for renewable energy. Solar energy is employed for both commercial and residential purposes^2^. Its benefits include being endless, pollution-free, abundant, silent, devoid of rotating parts, and capable of converting electricity effectively regardless of size. Although PV modules typically have a lifespan of approximately 25 years, several factors can affect their performance over time^2–6^. Since the PV installation occurs outdoors, it is exposed to environmental factors like solar irradiance, temperature, humidity, and physical stress, which have a significant impact on its performance over time^7–9^. The aging/degradation of the module is one of the key factors that affect the reduction in the power supply capacity of the module^10^.

There are several types of degradation that can affect PV modules. They include: Potential-induced degradation (PID): This type of degradation is often caused by a voltage potential difference between the grounding system and the modules' conductive parts, leading to a leakage current that can damage the module over time^8,11,12^. The second type is the light-induced degradation results from exposure to light that cause degradation of PV modules. In this type the ultra violet (UV) light in particular can break down the encapsulant materials and cause discoloration of PV cells, which reduces efficiency. This is known also as photo-degradation^4,5,8^. Another type is the degradation due to environmental factors^8,10^: The major environmental factors that induce degradation in PV modules are temperature, sunlight, rain, wind, humidity, mechanical stress, and dirt/sand accumulation which cause physical damage to the module's components, leading to degradation. These factors often interact and combine to decrease solar panel efficiency and longevity over their lifetime. Proper module sealing, installation, and maintenance can help mitigate some of these environmental degradation effects.

Studies reveal that environmental conditions have a significant impact on the energy produced by PV systems. These factors lead to PV degradation: corrosion, discoloration, delamination, and breakage. Humidity degrades the adhesion material between the PV cell and contact metal causing corrosion and so current leakage. It also causes metal surface corrosion which increases the delamination between solar cells and encapsulating material. The ultraviolet rays cause discoloration of the encapsulant material which increases the optical transmission losses. Sand storms lead to abrasion of the module surfaces while lightning strikes affect the metallic structures of the PV modules^13,14^.

Scientists use various methods for detecting defects in PV modules, such as electrical characterization, electroluminescence (LE), visual inspection, thermal imaging, and electrical insulation tests^15^. In the electrical characterization method, the modules are electrically disconnected from the system, and individually measured the I–V curve of each module under natural sunlight using a curve tracer. Visual inspection is an essential tool for identifying different apparent defects, such as cell cracks and encapsulant discoloration. Recording abnormalities, even if they initially have a minimal electrical impact, is important to track defect evolution. In the electroluminescence test, modules are forward-biased with a current in the order of I_SC_^15^. The recombination of electron–hole pairs results in low-intensity emission. As the emitted radiation occurs near the IR range (between the wavelengths of 1000 nm and 1300 nm), a specialized IR camera is used to detect the emission, as described by^16^. Since EL tests require a dark environment, indoor testing is generally easier. However, it can be performed outdoors under certain field conditions^16^. Thermography inspection of PV modules is a technology that helps identify faults in solar power plants. The inspection is carried out using infrared cameras and measures the temperature changes of the equipment in the plant. However, visual inspection is a powerful tool and is the most effective and quickest method to identify causes of failure in a PV module.

Many studies have been conducted on mono-crystalline modules to determine their degradation rate in various places around the globe (summarized in Table 1). In^14^, B. Aboagye et al. investigated the degradation rate of mono-crystalline modules in different locations in Ghana. The authors reported that after five years of exposure to various climatic conditions, the degradation rates are roughly 0.76 and 1.39 percent per year for dry equatorial and interior Savannah climates, respectively. The author attributed the high degradation rate of the interior Savannah climate to a higher temperature rate and dust accumulation than in dry equatorial areas. Likewise, in^17^, the authors deduced that the mean degradation rates of mono-crystalline, multi-crystalline, amorphous silicon (a-Si) modules are 1.37, 1.44, 1.67 percent per year, respectively. The climate type in Ghana is generally tropical and humid with high temperatures throughout the year. Therefore, the author reported faster degradation rates in Ghana than the standard warranty rates. Gyamfi et al.^18^ analyzed the power degradation rates of multi-crystalline silicon PV modules from 11 different manufacturers that were installed for 5 to 9 years in Kumasi, Ghana. Kumasi has a warm and humid climate, with a semi-deciduous forest. They found that under these weather conditions, the power degraded at rates between 0.79 and 1.67% per year. Piliougine et al.^19^ analyzed the degradation of single-crystalline silicon modules after 21 years in the field in Spain. The PV power is degraded annually by 0.9%. The author reported that the degradation occurs mainly due to a significant increase in the series resistance happened due to the corrosion level of the bus bars and interconnection ribbons. Another study was conducted by Lillo-Sánchez et al.^20^ after 22 years of PV installation in Seville, Spain. It has a subtropical and Mediterranean climate characterized by cold, wet winters and hot, dry summers. The peak power is deteriorating at a rate of 1.4 percent per year. In^21^, Raghuraman et al. conducted outdoor tests on three different PV module technologies (mono-Si, poly-Si, and a-Si) at Arizona State University, which has hot-arid climatic conditions. The authors found that the max power decreases by 0.4% to 0.5% per year for mono-Si technology after 4 years of outdoor exposure. They also found that the maximum power declined by 0.53% per year for ploy-Si modules, and from 1.16 to 3.52% per year for a-Si modules. An analysis of different test modules is presented in^22^ by Campbell et al. to study their performance in various countries over one year of testing. The test includes the USA and Germany. The authors concluded that the average degradation rates of mono-crystalline modules are 1 and 1.25% per year for the USA and Germany, respectively. While the average degradation rates of multi-crystalline modules are 1.2 and 2.1%/ year, 1.0 and 1.1%/ year for the USA and Germany, respectively. In the USA, mono-Si modules were found to be more reliable. While the multi-crystalline modules PV system in Germany was more effective than in USA.

**Table 1:** A literature review of PV modules degradation rates analysis.

Location	Test duration (Years)	Module technology	${{\varvec{d}}{\varvec{P}}}_{{\varvec{m}}{\varvec{a}}{\varvec{x}}}$ (%/year)	Reference
Ghana	5	Mono c-Si	0.76, 1.39	Aboagye et al.^14^
Ghana	5–10	Mono c-Si Multi c-Si a-Si	1.37 1.44 1.67	Aboagye et al.^17^
Ghana	5–9	11 different manufacturers	0.79–1.67	Gyamfi et al.^18^
Spain	21	Mono c-Si	0.89	Piliougine et al.^19^
Spain	22	Mono c-Si	1.4	L. Lillo-Sánchez et al.^20^
USA	2.4–4 2.4—2.7 2.7—6.7	Mono c-Si Multi c-Si a-Si	0.4–0.5 0.53 1.16–3.52	Raghuraman et al.^21^
USA	1	Mono c-Si Multi c-Si	0.9, 1.1 1.2, 2.1	Campbell et al.^22^
Germany	1	Mono c-Si Multi c-Si	1.3, 1.2 1.0, 1.1	Campbell et al.^22^
USA	11	Mono c-Si	4.39	Reis et al.^23^
Australia	1.3 1.3, 1.5 1.3 1.5	Mono c-Si Multi c-Si 3j a-Si CIS (LGBC) c-Si	1.03 1.01, 1.04 1.33 1.24 1.006	A. Carr et al.^24^
Italy	22	Mono c-Si	0.67	Dunlop et al.^25^
Singapore	10	Mono c-Si Multi c-Si CIS		W. Luo et al.^26^
Brazil	15	Mono c-Si	0.7	Fonseca et al.^15^
India	15	Mono c-Si	0.5	Kirmani et al.^27^
Algeria	11 9 9 12	Mono c-Si Mono c-Si Mono c-Si Multi c-Si	1.55 0.9 1.79 1.28	Sadok, et al.^28^
Algeria	11 20	Mono c-Si Mono c-Si	1.5 1.75	Sadok et al.^29^
Morocco	3	Mono c-Si	2.22, 4.12	Hajjaj et al.^30^
India	22	Mono c-Si	1.9	Rajput et al.^31^
India	22	Mono c-Si	1	Rajput et al.^32^

Another study in the USA was conducted by Reis et al.^23^ to measure the performance of mono-crystalline PV modules exposed to a cold marine environment over 11 years of employment. The authors reported a degradation rate of 0.399% per year in maximum power caused mainly by a decrease in short-circuit current. For more than a year, Carr et al.^24^ measured the performance of five different photovoltaic modules in Perth, Western Australia's temperate climate. The study examined five different module types: crystalline silicon (c-Si), laser grooved buried contact (LGBC) c-Si, polycrystalline silicon (p-Si), triple junction amorphous silicon (3j a-Si) and copper indium diselenide (CIS). The annual degradation rates calculated for the five module types were: 1.03% for c-Si, 1.01 to 1.04% for LGBC c-Si, 1.33% for p-Si, 1.24% for 3j a-Si, and 1.006% for CIS. The polycrystalline silicon modules showed the highest annual degradation rate, while the copper indium diselenide modules degraded the slowest. Over the test period, the standard deviation for the STC testing conditions is less than 1%. In^25^, Ewan D. Dunlop et al. measured and tested the characteristics of 40 silicon-based photovoltaic solar modules originating from six different manufacturers at the European Solar Test Installation after 20–22 years of continuous outdoor weathering. The results indicate that the degradation rate of mono-crystalline modules is about 0.67% per year. The authors mentioned that degradation and lifetime performance is dependent on the initial photon degradation and material aging. Luo et al.^26^ presented a case study of photovoltaic (PV) module failure rates after more than ten years of operation in Singapore's tropical climate. Mono-crystalline module degradation rates revealed a drastic power reduction (more than 4% per year). The annual degradation rates of multi-crystalline silicon modules were 0.85% and 1.05% respectively. Meanwhile, the annual degradation rates of CIS modules were approximately 4.5% and 1.57%. The authors attributed the severe power degradation to a combination of metallization corrosion and encapsulant discoloration, which results in transmittance loss. José E. Ferreira et al.^15^ conducted a study to measure the degradation rate of crystalline silicon photovoltaic modules caused by outdoor exposure after 15 years of installation in Porto Alegre, Brazil (characterized by hot summer and a humid temperate climate). The analysis showed that the average annual rate of degradation is 0.7% caused by the decrease of short circuit current. In^27^, Sheeraz Kirmani et al. analyzed long-term monitoring data to determine the degradation rates of crystalline modules after 15 years of field exposure in India, which was reported to be 0.5% per year. Two studies were conducted by Sadok, et al.^28,29^; one was for assessing the degradation of PV modules and detecting possible defects by a visual inspection method. The average annual power degradation rate of mono-crystalline PV modules is around 1.55% after 11 years of outdoor operation. While the average degradation rate of multi-crystalline PV modules is 1.28%/ year after 12 years of outdoor exposure. The other study is to assess the behavior of PV modules of different technologies after long-term exposure in the Saharan region of Algeria. The analysis showed a degradation rate of 1.75% per year after 20 years of field exposure. The Algerian Saharan climate is characterized by scorching summers, cold winters, low humidity, and sand storms. The authors reported that the main causes of power degradation are encapsulant discoloration, delamination, and burn marks. Hajjaj et al.^30^ performed a study to assess the performance decline of a photovoltaic system after three years of operation under harsh atmospheric conditions at the Green Energy Park research facility in Morocco. The annual power degradation rates are 2.22% and 4.12%. The authors attributed the severe energy drop to the presence of breakages and cracks at the module cells caused by high soiling rates and frequent cleaning events. Rajput et al.^31^ performed a degradation analysis of mono-crystalline PV modules after 22 years of outdoor exposure to the Indian climate. The analysis revealed a 1.9% power degradation rate per year. The authors identified the degradation in short circuit currents as the primary cause of degradation. Another analysis was carried out by Pramod et al.^32^, to assess the performance of PV mono-crystalline modules after 22 years of field exposure in India. The degradation rate is 1% in the maximum power.

This paper evaluates the performance of 24 mono-crystalline PV modules after 25 years of outdoor installation. The 1.8 kWp PV modules, installed on the rooftop of the electronics research institute (ERI) in Cairo, Egypt, are connected into six strings in parallel, with four modules in series in each string. Each PV module has a 75 W output, a maximum current of 4.4 A, and a maximum voltage of 17 V. This system is assessed using a range of performance indicators, such as energy yield, performance ratio, and efficiency. Module performance was evaluated by visual inspection and by measuring I–V curves outdoors under natural sunlight conditions using a solar simulator and I–V curve tracer. I–V curves were measured and translated to standard test conditions of 1000 W/m^2^ irradiance and 25 °C module temperature.

The paper is organized as follows: Section. “Methodology” presents the methodology of system installation, measurement, mathematical modeling, and also PV module parameter extraction under degradation. Section “Results and discussion” explains the obtained results of module visual inspection and parameters characterization. Finally, Sect “Conclusion” presents the conclusions and recommendations.

## Methodology

### Experimental setup and measurements

**Figure 1:**
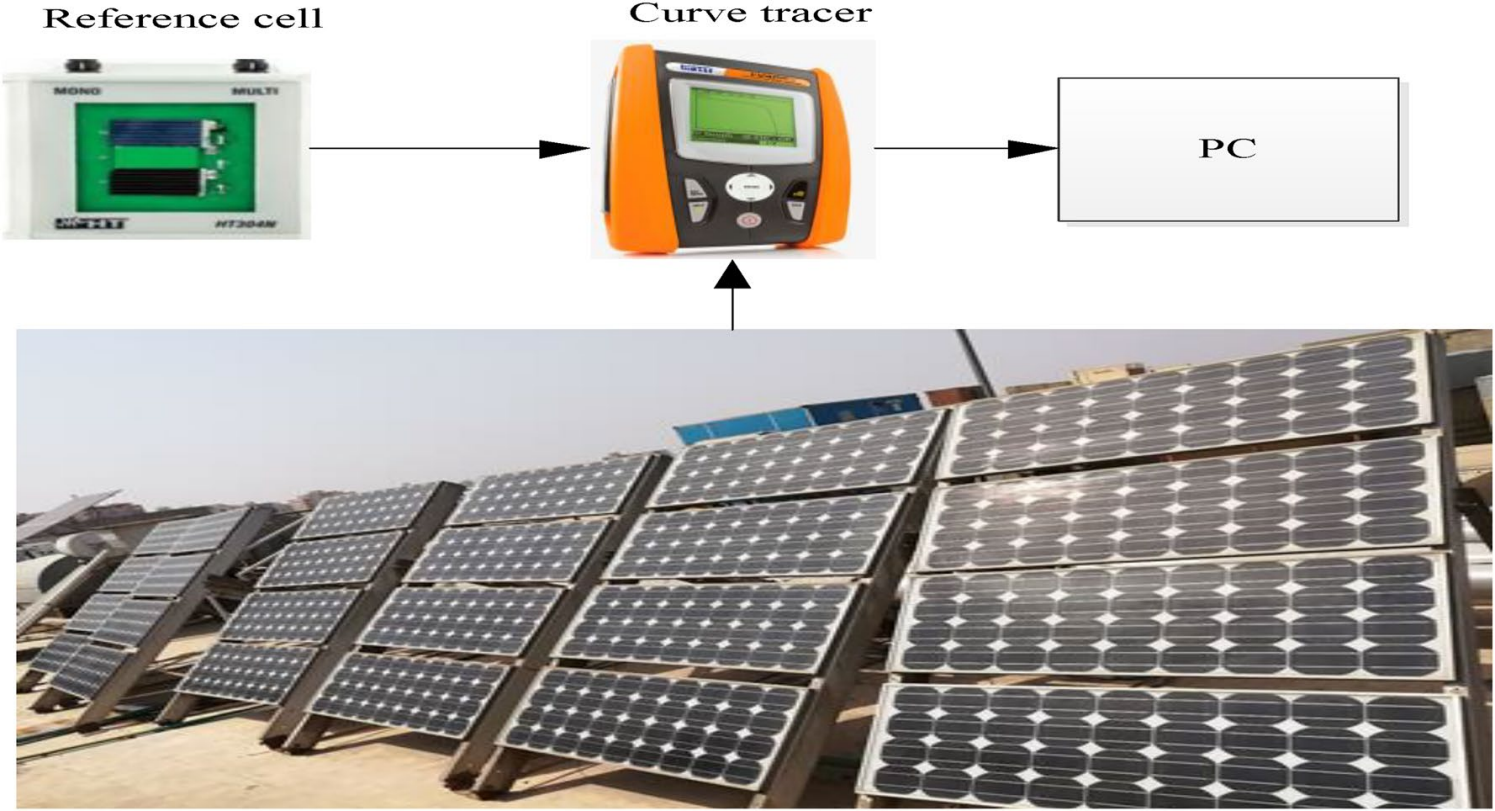
Experimental setup of PV modules under test.

**Figure 2:**
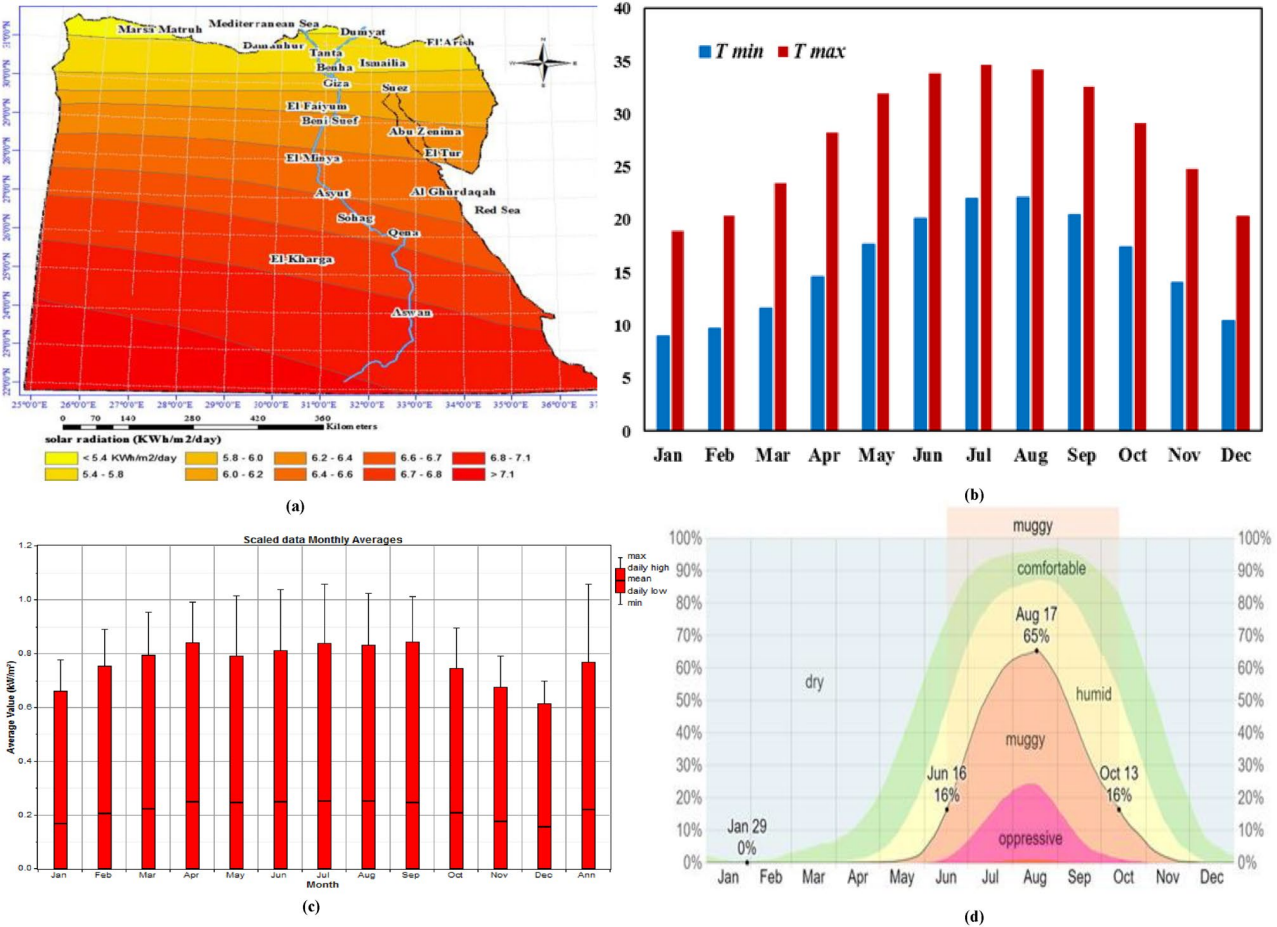
(**a**) Egypt solar radiation map, (**b**) Air temperature variation, (**c**) Monthly average solar irradiance, and (**d**) Percentage of time spent at various humidity comfort levels^34^.

Over the past 25 years, 24 modules have been installed on the rooftop of the electronics research institute (ERI) in Cairo, Egypt. The city of Cairo is located at 30° 1' latitude and 31° 14' longitude. Twenty-four PV modules are connected into six strings in parallel, with four modules in series in each string. The specific arrangement of the PV array was intended to supply a particular load at the PV Cells Department, ERI, following its installation. Nonetheless, for the purposes of this analysis, each module was tested individually, and the measurements were repeated for all 24 of them. By testing each module individually, we aimed to obtain accurate results about their individual performance characteristics. The modules were measured after 25 years in the field. Each PV module has a 75 W output, a maximum current of 4.4 A, and a maximum voltage of 17 V. Table 2 lists the detailed specifications of the PV module. Figure 1 shows the equipment used for the analysis, which includes the PV array under test, an I–V curve tracer to measure the parameters of the PV modules, a reference cell, and a personal computer. As shown in Fig. 1, modules are installed on a steel rack facing south with a 30° slope from the horizontal. The annual average solar radiation is 5.01 kWh/m^2^/day^33^. Cairo's average ambient temperature is 22.01 °C, the average relative humidity for the year is 54%, and the average wind speed over the year is 2.07 m/s^33^. June 16 to October 13 is the most humid period in Cairo, with at least 16% of those days being muggy, oppressive, or miserable. August is the month with the most humid days in Cairo (19.4 days). January has the fewest humid days in Cairo, with almost zero muggy days. Figure 2a–d shows the solar radiation map of Egypt, the annual average temperature and solar irradiance, and humidity level for Cairo^33,34^.

**Table 2:** Specification of SP75 solar module.

Electrical parameters	
Maximum power rating ${{{P}}}_{{{m}}{{a}}{{x}}}$ [Wp]	75
Maximum current ${{{I}}}_{{{m}}{{r}}}$ [A]	4.4
Maximum voltage ${{{V}}}_{{{m}}{{r}}}$ [V]	17.0
Short-circuit current ${{{I}}}_{{{s}}{{c}}}$ [A]	4.8
Open-circuit voltage ${{{V}}}_{{{o}}{{c}}}$ [V]	21.7
Thermal parameters	
${{N}}{{O}}{{C}}{{T}}$[°C]	45 ± 2
Temp. coefficient: short-circuit current $\boldsymbol{\alpha }{{i}}$	2.06 mA/°C
Temp. coefficient: open-circuit voltage ${{\beta}}$	− 0.077 V/°C

To ensure accurate data recording, the surfaces of the mounted PV modules are cleaned before taking measurements. So, the study does not account for the potential impact of dust accumulation on the PV module measurements. Additionally, the PV array is installed on a flat surface without facing any shading obstacles. As a result, the potential effects of shading on the analysis are not considered. According to the IEC 60904-1 standard (IEC, 2006)^35^, I–V characteristics were measured under standard test conditions (STC); 1000W/m^2^ irradiance, an air mass of 1.5, and an ambient temperature of 25 °C. The PV module electrical parameters were determined using a SOLAR I–V400w curve tracer with a measurement range of 15–1000 V, 1–15 A, and 20–100 °C. During the testing of a PV module, the I–V tracer is used to record various parameters. These recordings are then transferred to a PC for further analysis. Additionally, a reference cell is connected to the I–V tracer and is fixed in the same orientation as the PV module. This allows for the measurement of the solar radiation on the PV plane. In Table 3, the errors and precision of the I–V curve measurements taken with the SOLAR-I–V400w I–V tracer are presented^36^. In this paper, we analyzed the long-term performance degradation of PV modules through visual inspection of the modules, measurement of current–voltage (IV) curves normalized to STC, calculation of annual degradation rates, and estimation of PV parameters after 25 years of outdoor exposure. We have adjusted the I–V curves of the PV modules, which were measured in the field, to Standard Test Conditions (STC) to better estimate the rates of degradation.

**Table 3:** Accuracy in I–V tracer measurements.

Item	Range	Resolution	Accuracy
Temperature	–20:100 °C	0.1 °C	± (1% rdg + 1 °C)
Solar radiation (With reference cell)	1:100 mV	0.1 mV	± (1% rdg + 5dgt)
Power	50:99,999	1 W	± (1% rdg + 1dgt)
Voltage	15:99.9	0.1 V	± ( 0.5% rdg + 2dgt)
Current	0.1:15	0.01 A	± (1% rdg + 2dgt)

For each PV module, the I–V curves were measured individually under natural sunlight, following the guidelines of the standard IEC 60904-1 and ensuring that all modules were completely clean. The module temperature was recorded, and the global irradiance was measured using a reference cell. To reduce measurement and conversion errors, all measurements were taken within an hour of solar noon. The experimental I–V curves were then converted to the standard condition using mathematical modeling described in the following section, and implemented using MATLAB software.

### Mathematical modeling

For the I–V data translation method, calculation, and data analysis of procedures for temperature and irradiance corrections to measured I–V characteristics, a modified version of IEC 60891:2021^37^ (Procedures for temperature and irradiance corrections to measured I–V characteristics.) is described as follows^38^^39^:

1 $${I}_{SC2}={I}_{SC1}\left[1+\alpha i\left({T}_{2}-{T}_{1}\right)\right]\frac{{G}_{2}}{{G}_{1}}$$

2 $${V}_{OC2}={V}_{OC1}\left[1+\gamma ln\frac{{G}_{2}}{{G}_{1}}+\beta \left({T}_{2}-{T}_{1}\right)\right]$$

3 $${I}_{2}={I}_{1}\left(\frac{{I}_{SC2}}{{I}_{SC1}}\right)$$

4 $${V}_{2}={V}_{1}+\left({V}_{OC2}-{V}_{OC1}\right)+{R}_{s}\left({I}_{1}-{I}_{2}\right)$$

5 $${P}_{max1}=\left(\frac{{G}_{1}}{{G}_{2}}\right)\left[\frac{{P}_{max}}{1+\gamma \left({T}_{2}-{T}_{1}\right)}\right]$$

6 $${I}_{max1}=\frac{{I}_{max2}\left(\frac{{G}_{1}}{{G}_{2}}\right)}{\left(1+\alpha i\left({T}_{2}-{T}_{1}\right)\right)}={I}_{max2}\left(\frac{{I}_{SC1}}{{I}_{SC2}}\right)$$

7 $${V}_{max1}=\frac{{V}_{max2}}{\left(1+\beta \left({T}_{2}-{T}_{1}\right)\right)}={V}_{max2}\left(\frac{{V}_{OC1}}{{V}_{OC2}}\right)$$

8 $${FF}_{1}=\left[\frac{{P}_{max1}}{{I}_{SC1}{V}_{OC1}}\right]$$

where $I$ is current (A), ${I}_{SC}$ is short-circuit current (A), $G$ is solar irradiance (W/m^2^), $T$ is module temperature (C), $V$ is voltage (V) and ${V}_{OC}$ is open-circuit voltage (V). subscripts 1 and 2 refer to the measured, and values at reference conditions respectively. $\beta$ is the temperature coefficient of ${V}_{OC}$, $\gamma$ and $\alpha i$ are the irradiance correction factor, and temperature coefficient for current, respectively, and ${R}_{s}$ is series resistance (Ω).

The degradation rate of each PV module parameter was estimated analytically using the following equation^40^:

9 $${R}_{d}\left(X\right)=\left(1-\frac{X}{{X}_{o}}\right),$$

Where, $$\left\{\begin{array}{c}X=\left[ {P}_{max}\,{I}_{m}\,{ V}_{m}{ I}_{SC }{ V}_{OC} FF\,\eta \right]\\ {X}_{o}=\left[ {P}_{maxo}\,{I}_{mo}\,{ V}_{mo}{ I}_{SCo }{ V}_{OCo}\,{FF}_{o} {\eta }_{o}\right]\end{array}\right\}$$

10 $${R}_{d}\left(X\right)\%=\frac{{R}_{d}}{\Delta N}$$

where ${R}_{d}$ is the degradation rate, ${X}_{o}$ is the manufacturer's reference value for the parameters under STC and $X$ is the value after degradation, and $N$ (years) is the time of exposure under actual conditions.

### Performance methodology

The performance of PV systems is often significantly affected by geography and climate^41^. The performance analysis parameters provide the overall performance of the PV system with energy yield, solar insolation, and overall system losses. The most widely used parameter for assessing the performance of a PV system under field-exposed conditions is the Performance Ratio ($PR$), which is a technique for determining the PV system's actual efficiency^42,43^.

A PV system's performance is typically assessed using a range of performance indicators, such as energy yield, performance ratio, and efficiency. The performance ratio ($PR$) calculates the overall effect of losses on the system's rated output and indicates how close it is to ideal performance under actual conditions. The $PR$ is used to compare modules that get different levels of irradiation due to geographical position or PV tilt. The performance ratio of the PV units, $PR$, is calculated by^44,45^:

11 $$PR\left(\%\right)=\frac{Y}{{Y}_{r}} . 100$$

where $Y$ is the energy yield, which indicates how long PV modules should be able to operate at their rated power. The output of a PV system, normalized by its rated capacity, is known as the energy yield. It specifies the number of hours the PV system should operate at rated power each day to generate the same amount of energy as formerly measured^46^. It can be determined using^47–49^:

12 $$Y=\frac{E}{{P}_{\mathrm{max}STC}}$$

where $E$ represents the energy output of the tested photovoltaic modules. It is calculated based on the I–V measurements, while ${P}_{\mathrm{max}STC}$ denotes the maximum power measured at STC, provided in the module datasheet.

The reference yield, ${Y}_{R}$, is the ratio of total in-plane solar radiation ($G$), measured by the reference cell, to the array reference irradiance ${G}_{r}$ (typically 1 kW/m^2^). It's a measurement of the theoretical energy available at a given location over a given period, calculated as^46^:

13 $${Y}_{R}=\frac{G}{{G}_{r}}$$

The annual loss (W) is then:

14 $${P}_{loss}=\%{{R}_{d}}_{Pmax } \times {P}_{MaxRef}$$

### PV module parameters estimation

**Figure 3:**
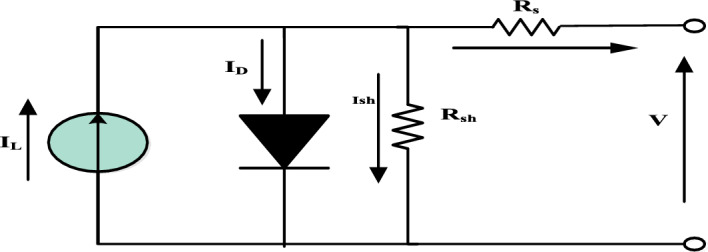
SDM Solar cell equivalent circuit.

The behavior of PV cells is described by an equivalent circuit model using a Single Diode Model (SDM) of a PV module. This model is commonly used to simulate PV cells and is shown in Fig. 3. The variation in the internal parameters such as *I*_*pv*_*, I*_*o*_*, A, *${R}_{s}$*, and* ${R}_{sh}$ of the PV modules in the field exposed conditions has been investigated in this study. The parameters extraction technique is employed to find the model parameters. The input electrical parameters for the present parameters extraction techniques were monitored under outdoor conditions using an experimental setup. Since the present technique utilizes the input parameters at STC (Irradiance is 1 kW/m^2^, module temperature is 25 °C and air mass is AM1.5), the monitored electrical parameters are translated to the STC using the methodology described in^43^. Five nonlinear equations are derived using I–V characteristics to find the PV model parameters. Generally, the PV module I–V characteristic is passed through the three points of the STC: short circuit current, open circuit voltage, and maximum power point current and voltage. However, the five-parameters model is the most used because it is a good compromise between precision and simplicity^50^.

The output current is determined by the following equation:

15 $$I={I}_{L}- {I}_{o}\left[{e}^{\frac{\left(V+{R}_{s} I\right)}{{v}_{t}}}-1\right]- \frac{V+{R}_{s}I}{{R}_{sh}}$$

${v}_{t}$ is the thermal voltage and is defined as^51^:

16 $${v}_{t}=\frac{kAT}{q}$$

The series resistance ${R}_{s}$ is defined as:

17 $${R}_{s =} \frac{{V}_{oc}}{{I}_{mp}}+ \frac{{v}_{t}}{{I}_{m}} ln\left(\frac{{v}_{t}}{{v}_{t}+ {V}_{m}}\right)- \frac{{V}_{m}}{{I}_{m}}$$

The maximum power voltage can thus be obtained:

18 $${V}_{m}= \left[{v}_{t }\mathrm{ln}\left(\frac{{I}_{L}+ {I}_{o}- {I}_{m}}{{I}_{o}}\right)\right]- \left({R}_{s }{I}_{m}\right)$$

The proposed method provides an initial estimation of ${R}_{sh}$ that can be obtained from the relation of the maximum power as follows:

19 $${I}_{m}={I}_{L}- {I}_{o}\left[{e}^{\frac{\left({V}_{m}+{R}_{s} {I}_{m}\right)}{{v}_{t}}}-1\right]- \frac{{V}_{m}+{R}_{s}{I}_{m}}{{R}_{sh}}$$

From (19), ${R}_{sh}$ is extracted which is worth:

20 $${R}_{sh}=\frac{{V}_{m}\left({V}_{m}+{R}_{s}{ I}_{m}\right)}{{V}_{m}{I}_{L}-{V}_{m }{I}_{o}\left({e}^{\frac{{V}_{m}+{R}_{s }{I}_{m}}{{v}_{t}}}-1\right)-{P}_{max}}$$

The new values of *I*_*o*_ and *I*_*L*_ will be:

21 $${I}_{o}=\frac{{I}_{sc}\left(1+\frac{{R}_{s}}{{R}_{sh}}\right)-\frac{{V}_{oc}}{{R}_{sh}}}{{e}^{\frac{{v}_{oc}}{{v}_{t}}}-{e}^{\frac{{R}_{s} {I}_{sc}}{{v}_{t}}}}$$

22 $${I}_{L}={I}_{o}\left[{e}^{\frac{{V}_{oc}}{{v}_{t}}}-1\right]+\frac{{V}_{oc}}{{R}_{sh}}$$

The algorithm has been tested using MATLAB software since it is an iterative algorithm that uses simple equations that are easily solved as indicated in the pseudo code below.

## Pseudo code of parameter estimation algorithm:

1. Input module datasheet*: *${I}_{sc}$*, *${V}_{oc}$*, *${I}_{mr}$*, *${V}_{mr}$*, A, max. iter, tolv, toli.*
2. Calculate the initial values of ${v}_{t}$*, *${R}_{s}$*, *${I}_{o}$*,* ${I}_{L}$.
3. Calculate ${V}_{m}$, then check if $$\left\{\begin{array}{c}{V}_{m}>{V}_{mr}, A=A-0.01\\ {V}_{m}<{V}_{mr}, A=A+0.01\end{array}\right.$$
4. Calculate new values of (*I*_*o*_*, I*_*L*_*, *${v}_{t}$*,* ${V}_{m}$), then check *errv* = ${V}_{m}$*-*${V}_{mr}$,
5. if *errv* > *tolv & iter* < *max.iter* $$\left\{\begin{array}{c}yes, then\,return\,to\,step\,3 \\ no, Rs=Rsnew , A=Anew\end{array}\right.$$  
6. Calculate new values of (*I*_*o*_*, I*_*L*_*,* ${v}_{t}$) using $Rsnew$, $Anew$ from the previous step. Then calculate ${R}_{sh}$.
7. Calculate ${I}_{m}$, then check if $$\left\{\begin{array}{c}{I}_{m}>{I}_{mr}, {R}_{sh\,new}={R}_{sh}-0.1*iter\\ {I}_{m}<{I}_{mr}, {R}_{sh\,new}={R}_{sh}+0.1*iter\end{array}\right.$$  
8. Calculate new values of (*I*_*o*_*, I*_*L*_), then check *erri* = ${I}_{m}$*-*${I}_{mr}$*, if erri* > *toli & iter* < *max.iter* $$\left\{\begin{array}{c}yes, then\,return\,to\,step\,6 \\ no, End\end{array}\right.$$  
9. Print Final parameters.

## Results and discussion

### Visual and physical inspection

The visual inspection of the PV modules under test involved evaluating all PV system components, including:

- The front glass surface
- The back sheet
- Wiring and connectors
- Junction boxes
- Frames
- Bus bars
- Cell interconnects

**Figure 4:**
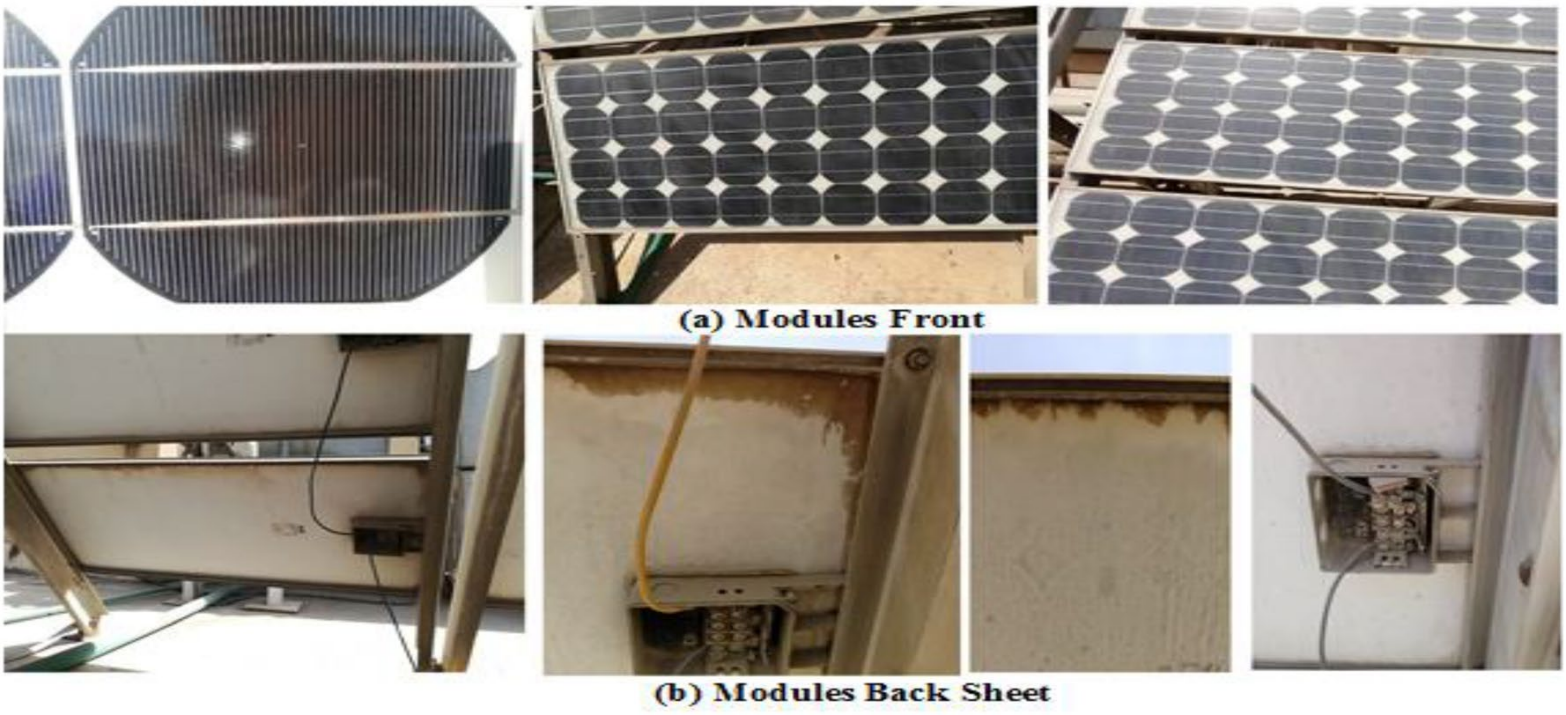
The visual inspection of PV modules.

The visual observation of the tested PV modules was resulted in the following: regarding the front glass surface, it is observed that all modules have smooth front glass; no damage or cracks were visible. As well, no wavy texture, chalking, burn marks, or other signs of damage were found on the back sheet. Regarding the wiring and connectors, there are no burns or brittleness in the wires or connectors. All module junction boxes are complete and firmly attached but all junction boxes of each module were opened; no signs of adhesion loss were found (all junction boxes are fixed tightly to PV modules indicating that the electrical terminals remained secure). The loss of adhesion causes the failure of electrical terminals which contributes to the failure of the PV module. The bottom section of the frames was dirty and had accumulated dust over the years, but there was no discoloration, corrosion, or evidence of frame adhesive was degrading. Bus bars and cell interconnect displayed no corrosion, discoloration, or metallization. The condition of the PV cells and cells interconnects of each module was found to be good. As indicated in Figs. 1 and 4, which emphasizes the findings of the visual inspection of PV modules, all modules are in very good condition. Despite this, some modules have minor discoloration, which interprets the highest degradation of some modules than others. Also, junction boxes are all in very good condition and connected tightly to their electrical terminals. Furthermore, Fig. 4B illustrates that there is discoloration at the encapsulation edges of minor modules, which is an indication of higher degradation rates compared to the other modules. This discoloration is observed on the back of the PV modules. Module No.5 exhibited the most severe discoloration affecting the front glass surface of the PV module as well as the edges of the encapsulant.

### Electrical characterization

**Figure 5:**
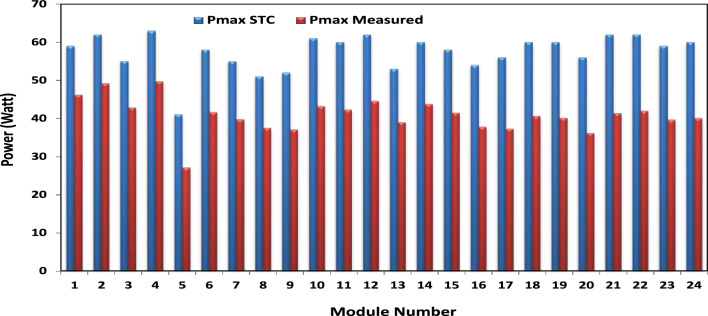
The measured and STC maximum powers for each module.

It investigated how electrical characteristics changed as the field aged from 1997 to 2022. Figure 5 depicts the measured maximum power and maximum power adjusted to STC of the 24 modules under test. The maximum power of module 5 has the lowest generated value, reaching 53% of its maximum, whereas module 4 has the highest value, reaching 87% of its maximum value after 25 years of outdoor operation. The visual inspection revealed signs of discoloration on the front glass surface and encapsulant edges of Module No. 5. This discoloration would have reduced the transmissivity of light into the PV module, explaining its comparatively lower performance parameters. The higher series resistance and reduced light transmission, due to the observed discoloration, combined to decrease the maximum current and fill factor for Module No. 5.

**Figure 6:**
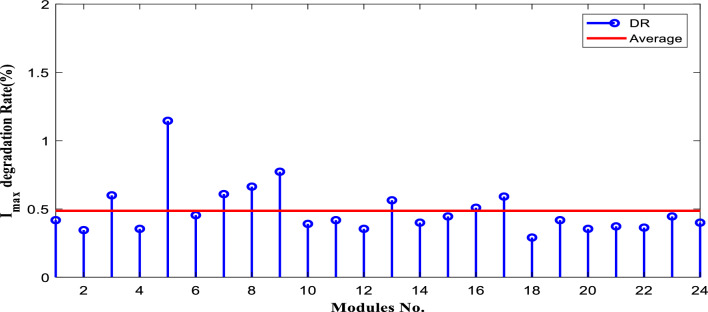
Annual degradation rate of maximum current.

**Figure 7:**
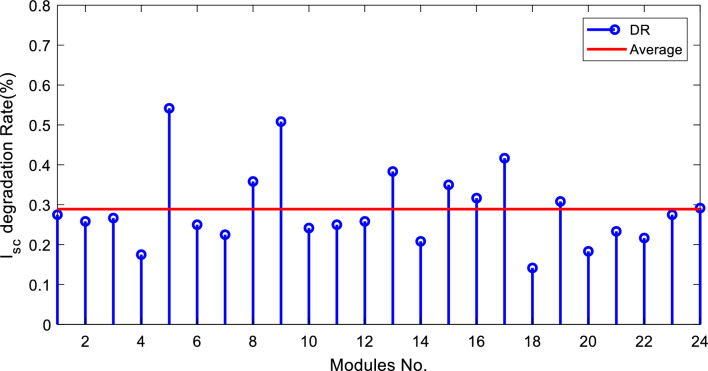
Annual degradation rate of short-circuit current.

**Figure 8:**
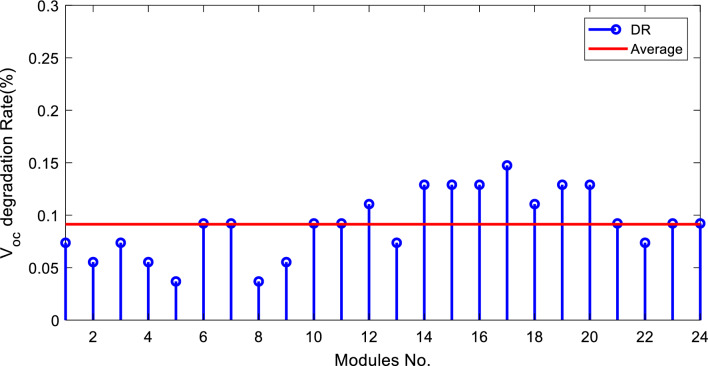
Annual degradation rate of module open-circuit voltage.

**Figure 9:**
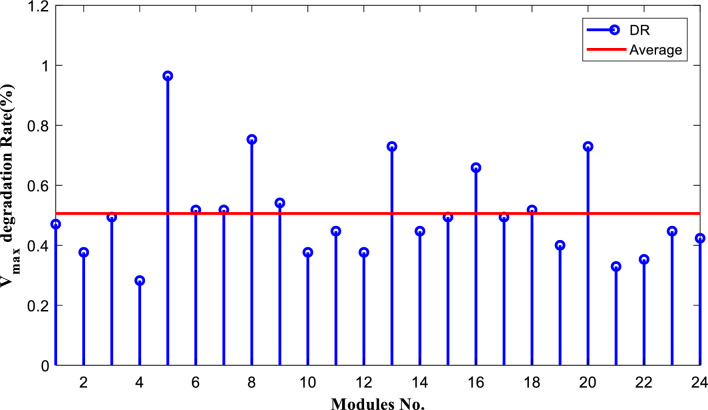
Annual degradation rate of module maximum voltage.

**Figure 10:**
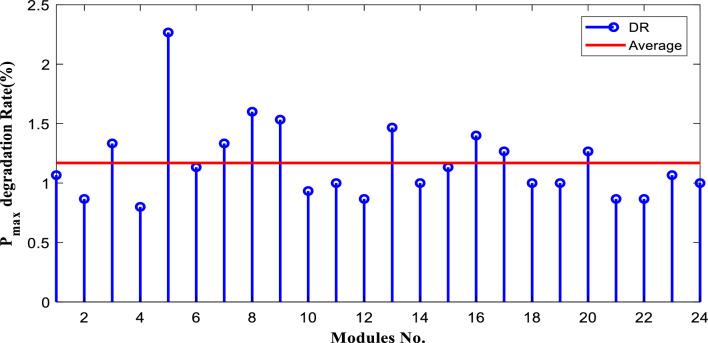
Annual degradation rate of module maximum power.

**Figure 11:**
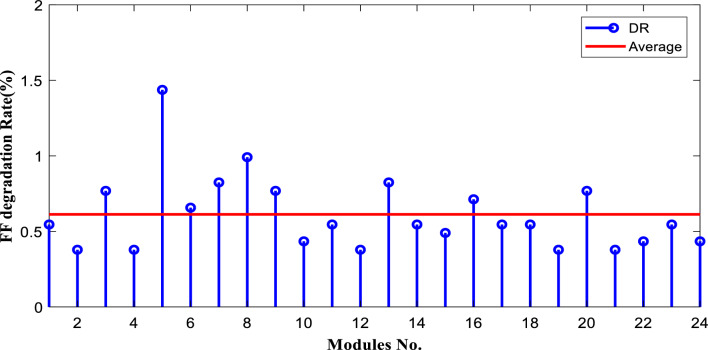
Annual degradation rate of module fill factor.

**Figure 12:**
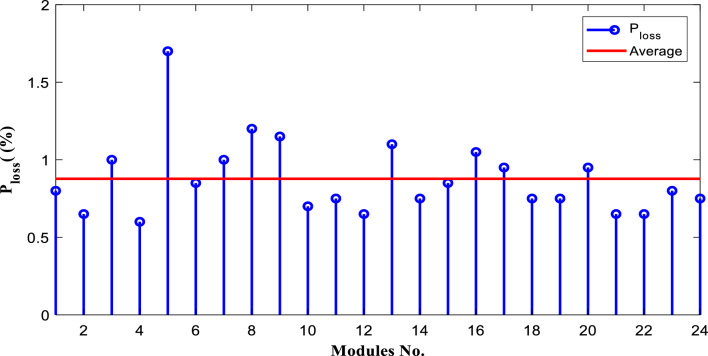
The annual power loss variation.

**Figure 13:**
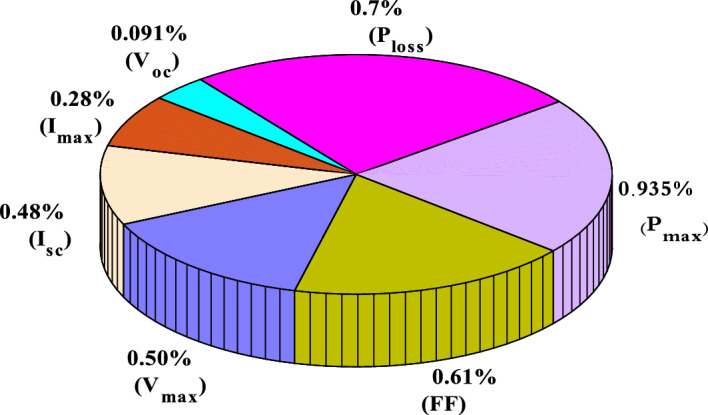
Degradation rate of mono-crystalline PV module SP 75 after 25 years of outdoor operation.

The degradation rates of PV parameters (${I}_{m}$*, *${I}_{sc}$, ${V}_{oc}$*, *${V}_{m}$*, *${P}_{max}$*, and* $FF$) are calculated for each module using Eqs. (9) and (10) based on the measured data after 25 years of field operation. The measured data was first converted to STC using Eqs. (1) to (8) as shown in Table 4, which gives a sample of the measured data. The PV module data sheet given in Table 2 was also used in the calculations. The degradation rates of ${I}_{m}$*, *${I}_{sc}$*, *${V}_{oc}$*, *${V}_{m}$*, *${P}_{max}$*, and *$FF$ are illustrated in Figs. 6 through 11, respectively. The annual degradation rate of ${I}_{m}$ varies in the range of 0.072% to 0.286%, given in Fig. 6. Figure 7 reveals that ${I}_{sc}$ has an annual degradation rate of 0.035% as a minimum value and 0.135% as a maximum value. As shown in Fig. 8, the ${V}_{oc}$ degradation rate varies within a range of 0.0092% to 0.0368% per year. As adopted in Fig. 9, the ${V}_{m}$ has an annual degradation rate of 0.0705% to 0.2411%, while ${P}_{max}$ records a 0.16% minimum annual degradation rate and a 0.453% maximum value of annual degradation rate as denoted in Fig. 10. Finally, $FF$ records annual degradation rates in ranges from 0.0947% to 0.359%, as in Fig. 11. Figure 12 gives the annual loss of power variation of the PV module under test according to Eq. (18) The average value of variation is 0.7%. Figure 13 summarizes the annual degradation rate of the mono-crystalline PV module SP 75 after 25 years of outdoor operation.

**Table 4:** Sample of the I–V curve tracer measurements.

Module	Rad	Ambient temp	ImaxSTC	VmaxSTC	IscSTC	VocSTC	PmaxSTC
1	795	27	3.94	15	4.47	21.3	59
2	804	28	4.02	15.4	4.49	21.4	62
3	793	27	3.74	14.9	4.48	21.3	55
4	798	28	4.01	15.8	4.59	21.4	63
5	735	29	3.14	12.9	4.15	21.5	41
6	744	29	3.9	14.8	4.5	21.2	58
7	754	28	3.73	14.8	4.53	21.2	55
8	770	27	3.67	13.8	4.37	21.5	51
9	740	27	3.55	14.7	4.19	21.4	52
10	727	27	3.97	15.4	4.51	21.2	61
11	732	26	3.94	15.1	4.5	21.2	60
12	737	27	4.01	15.4	4.49	21.1	62
13	763	28	3.78	13.9	4.34	21.3	53
14	752	27	3.96	15.1	4.55	21	60
15	729	27	3.91	14.9	4.38	21	58
16	719	27	3.84	14.2	4.42	21	54
17	694	28	3.75	14.9	4.3	20.9	56
18	697	29	4.08	14.8	4.63	21.1	60
19	687	30	3.94	15.3	4.43	21	60
20	678	28	4.01	13.9	4.58	21	56
21	688	29	3.99	15.6	4.52	21.2	62
22	700	28	4	15.5	4.54	21.3	62
23	697	28	3.91	15.1	4.47	21.2	59
24	687	28	3.96	15.2	4.45	21.2	60

The yearly average of $PR$ is 85.9%, the annual yield is 4.59 (h/d), and the reference yield is 5.35 (h/d). As a result, it can be stated that the performance of PV plants in outdoor environments diminishes over time. The time-series data of several performance parameters can be used to observe the PV system's performance decline trend.

After 25 years of operation, PV parameters estimation is carried out to determine the degradation effect of outdoor exposure on five module parameters (reverse saturation current (*I*_*o*_), light generated current (*I*_*g*_), ideality factor (*n*), series (${R}_{s}$) and shunt (${R}_{sh}$) resistance). Table 5 gives the estimation of PV parameters for each module under test. The three parameters named *I*_*o*_*, *${R}_{s}$*, and* ${R}_{sh}$ have been affected by the operation in an open environment. As a result, the shunt resistance can be used to calculate the solar cell's health index. Even though PV cells and modules are designed to reduce series resistance losses, ${R}_{s}$ steadily increase when exposed to environmental conditions. The increase in ${R}_{s}$ is due to metallic corrosion, which diminishes conductivity. An increase in ${R}_{s}$ does not affect ${V}_{oc}$, although they do diminish ${I}_{sc}$. The main cause of module performance decline has been recognized as an increase in series resistance. It is mostly caused by a decrease in electron production. Although extremely high values may also limit the short-circuit current, the major effect of series resistance is to diminish the fill factor. It reduces the maximum output power that can be achieved. It can be shown that the ${R}_{s}$ reduce the voltage output, fill factor, and so module efficiency by lowering the slope of the IV characteristics. The cell and metallization contact, the metallization and ribbon contact, and the ribbon and ribbon contact all have the potential to enhance series resistance.

**Table 5:** degradation effect on PV parameters under testing after 25 years of outdoor exposure.

Module No	*A*	*I*_o_*10^*–9*^	*I*_*L*_	${{\varvec{R}}}_{{\varvec{s}}}$	${{\varvec{R}}}_{{\varvec{s}}{\varvec{h}}}$
Mref	1.1	2.65	4.798	0.352244	942
M1	1.1	3.66	4.466	0.762224	962
M2	1.1	3.33	4.487	0.693759	955
M3	1.1	3.67	4.477	0.737256	975
M4	1.1	3.41	4.587	0.533243	966
M5	1.1	2.79	4.144	1.528386	982
M6	1.1	4.07	4.496	0.769516	968
M7	1.1	4.09	4.527	0.723179	977
M8	1.1	2.94	4.365	1.15285	972
M9	1.1	3.11	4.186	0.892019	970
M10	1.1	4.08	4.507	0.615582	963
M11	1.1	4.07	4.497	0.695078	964
M12	1.1	4.48	4.487	0.615008	957
M13	1.1	3.55	4.335	1.098493	964
M14	1.1	5.00	4.547	0.629844	967
M15	1.1	4.82	4.377	0.747689	955
M16	1.1	4.86	4.416	0.910076	966
M17	1.1	5.22	4.297	0.702292	965
M18	1.1	4.61	4.626	0.741664	963
M19	1.1	4.87	4.427	0.61967	959
M20	1.1	5.03	4.575	0.97045	964
M21	1.1	4.08	4.517	0.562031	962
M22	1.1	3.72	4.537	0.609756	963
M23	1.1	4.04	4.467	0.698563	965
M24	1.1	4.02	4.447	0.696601	958

Reducing the diode saturation current increases the open circuit voltage of the solar cell. As ${I}_{o}$ increases, the annual degradation of ${V}_{oc}$ also increases, as shown in Fig. 8. Module 5 has a minimum value of ${I}_{o}$, which corresponds to the minimum value of ${V}_{oc}$ degradation. The series resistance $({R}_{s })$ affects the cell’s power output. A reduction in series resistance will result in an increase in output power and also a deviation from the maximum power point. Module 5 has the highest series resistance value, which is related to the highest value of power loss annual degradation of a specific module as adopted in Fig. 12. Also, ${R}_{s}$ mainly affected ${I}_{sc}$; as given in Table 5, the modules with high values of ${R}_{s}$ have a high-value degradation rate in ${I}_{m}$, ${I}_{sc}$, and ${P}_{max}$ as indicated in Figs. 6, 7, and 10, respectively.

## Conclusion

Using the SOLAR I–V400w curve tracer, this study investigated the effects of real-world external conditions on the performance of solar modules after more than 25 years of exposure. Several translation procedures have been assessed in the literature and decided to adopt processes 1 and 2 of IEC 60891:2021 for the I–V conversion from field data to STC. MATLAB software has been used to implement these techniques. The results revealed a 0.93% annual average decline in ${P}_{max}$ for the studied 24 mono-crystalline PV modules. ${I}_{sc}$ loss has a yearly degradation rate of 0.288%, while $FF$ and ${V}_{oc}$ losses are 0.61% to 0.091% per year, respectively, and finally, the performance ratio obtained is 85.9%. The degradation impact on five module parameters (light-generated current, reverse saturation current, ideality factor, series resistance, and shunt resistance) was investigated while estimating these parameters. It was found that reverse saturation current, series resistance, and shunt resistance were the parameters affected by degradation. The overall experimental results show that Cairo's environmental conditions have no significant impact on PV performance. This is important because PV arrays continue to perform well, exceeding their expected lifetime, and the degradation rate after 25 years of operation is adequate.

## Data Availability

All data generated are included in the paper.
